# For more Mangomoments in health care!

**DOI:** 10.31744/einstein_journal/2026CE1486

**Published:** 2026-03-13

**Authors:** Alexsandro Tartaglia, Marcos Antônio Almeida Matos, Mary Gomes Silva, Felipe Reis

**Affiliations:** 1 Escola Bahiana de Medicina e Saúde Pública Salvador BA Brazil Escola Bahiana de Medicina e Saúde Pública, Salvador, BA, Brazil.; 2 Hospital Geral Roberto Santos Salvador BA Brazil Hospital Geral Roberto Santos, Salvador, BA, Brazil.

Dear Editor,

What does Mangomoments mean? In 2018, Kris Vanhaecht, a professor at Leuven University in Belgium, published a paper in the Lancet Oncology Journal titled "In search of Mangomoments".^(1)^ The title drew inspiration from a documentary filmed at a Belgian hospital featuring a patient in the intensive care unit (ICU) and a journalist. Upon awakening from a coma, the patient described the difficulty of lying motionless, staring at the ceiling, listening to her family during visits, and witnessing the care team's dynamics. Moved by her story, the journalist asked what could make her happy. She immediately replied that she would love to have a mango – just to taste one once again. Days later, the journalist returned with a mango and gave it to her as a gift. This simple action brought the patient to tears. Upon hearing this, Vanhaecht was touched yet puzzled, reflecting that no ICU health professional had asked this fundamental question: "What can we do to make her happier during this difficult time?"^(1,2)^

This story became the catalyst for Professor Vanhaecht's people-centered care initiative, "In search of Mangomoments." He defines these moments as small, unexpected gestures of kindness and leadership from health professionals toward patients and their families that can significantly enhance care quality.^(1)^ In addition, he emphasizes their value in the care experience for patients, family members, and health professionals.^(2)^ These moments occur during routine care activities and require minimal additional resources, time, or energy.^(1,2)^

For health professionals to provide Mangomoments, they must have opportunities to do so, making management support essential. Leadership must facilitate opportunities and foster health care innovations to advance patient-centered care. Positive care experiences enhance health professionals’ resilience and workplace engagement.^(1,3)^

This positive dynamic establishes trust and greater tolerance during challenging care experiences. Furthermore, fostering open communication among patients, families, and health professionals—addressing concerns and explaining care processes—develops psychological safety and improves institutional climate.^(3)^ Identifying individual patient needs enables health professionals to respond more effectively.^(4)^

Health care encompasses beautiful and difficult moments. Positive situations should be shared widely and quickly.^(2)^

Notably, Mangomoments must align with safety standards. Joy-inducing actions should respect clinical and institutional protocols.^(3)^ Many health professionals across Brazil have initiated Mangomoments. We must promote these to inspire health organizations nationwide to adopt them. Incorporating these moments into daily practice makes health care more humane and less burdensome.

## Data Availability

The underlying content is contained within the manuscript.
